# Decreased Brain Serotonin in *rbfox1* Mutant Zebrafish and Partial Reversion of Behavioural Alterations by the SSRI Fluoxetine

**DOI:** 10.3390/ph17020254

**Published:** 2024-02-16

**Authors:** Maja R. Adel, Ester Antón-Galindo, Edurne Gago-Garcia, Angela Arias-Dimas, Concepció Arenas, Rafael Artuch, Bru Cormand, Noèlia Fernàndez-Castillo

**Affiliations:** 1 Departament de Genètica, Microbiologia i Estadística, Facultat de Biologia, Universitat de Barcelona, 08028 Barcelona, Spain; 2 Centro de Investigación Biomédica en Red de Enfermedades Raras (CIBERER), Instituto de Salud Carlos III (ISCIII), 28029 Madrid, Spain; 3 Institut de Biomedicina de la Universitat de Barcelona (IBUB), 08028 Barcelona, Spain; 4 Institut de Recerca Sant Joan de Déu, 08950 Esplugues de Llobregat, Spain; 5 Clinical Biochemistry Department, Institut de Recerca Sant Joan de Déu, Hospital Sant Joan de Déu, 08950 Esplugues de Llobregat, Spain

**Keywords:** *RBFOX1*, psychiatric disorders, zebrafish, fluoxetine, serotonin

## Abstract

*RBFOX1* functions as a master regulator of thousands of genes, exerting a pleiotropic effect on numerous neurodevelopmental and psychiatric disorders. A potential mechanism by which *RBFOX1* may impact these disorders is through its modulation of serotonergic neurotransmission, a common target for pharmacological intervention in psychiatric conditions linked to *RBFOX1*. However, the precise effects of *RBFOX1* on the serotonergic system remain largely unexplored. Here we show that homozygous *rbfox1^sa15940^* zebrafish, which express a shorter, aberrant *rbfox1* mRNA, have significantly reduced serotonin levels in telencephalon and diencephalon. We observed that the acute administration of fluoxetine partially reverses the associated behavioural alterations. The hyperactive phenotype and altered shoaling behaviour of the *rbfox1^sa15940/sa15940^* zebrafish could be reversed with acute fluoxetine exposure in the Open Field and the Shoaling test, respectively. However, in the other paradigms, hyperactivity was not diminished, suggesting a distinct intrinsic motivation for locomotion in the different paradigms. Acute fluoxetine exposure did not reverse the alterations observed in the aggression and social novelty tests, suggesting the involvement of other neurological mechanisms in these behaviours. These findings underscore the importance of investigating the intricate working mechanisms of *RBFOX1* in neurodevelopmental and psychiatric disorders to gain a better understanding of the associated disorders along with their pharmacological treatment.

## 1. Introduction

The *RNA Binding Fox-1 Homolog 1 (RBFOX1)* gene encodes a neuronal splicing factor with a pleiotropic effect on numerous neurological, neurodevelopmental, and psychiatric disorders [1,2,3,4,5,6,7]. While functioning primarily as a splicing regulator, RBFOX1 has also been shown to stabilise target mRNA levels in the cytoplasm, and it controls extensive gene networks implicated in various neurodevelopmental and psychiatric disorders [1,2,8]. *RBFOX1* copy number variants have been associated with epilepsy, autism spectrum disorder (ASD), schizophrenia (SCZ), mild to severe global developmental delay, and intellectual disability [3,4,7]. It has been identified as a candidate gene for aggression in human and rodent studies, directly and through the regulation of other aggression-related genes [4,5]. Furthermore, in a recent cross-disorder study investigating the genetic architecture of eleven psychiatric disorders, including attention-deficit/hyperactivity disorder (ADHD), ASD, anxiety disorders, bipolar disorder, major depressive disorder (MDD), obsessive–compulsive disorder (OCD), and SCZ, among others, *RBFOX1* emerged as an independent associated locus in genome-wide association study (GWAS) across all eleven psychiatric traits [9]. It was also identified in a subset of internalising disorders and as a higher-order transdiagnostic psychopathology factor. This aligns with a previous GWAS meta-analysis across eight psychiatric disorders where *RBFOX1* was recognised as the second most pleiotropic locus [6]. Recently, the serum levels of RBFOX1, along with transcription factor 4 (TCF4), have been proposed as potential biomarkers for depression due to their substantially increased levels in unmedicated patients with MDD [10].

Given its significant role in neurodevelopment and psychiatric disorders, the study of *RBFOX1* has garnered considerable interest in recent years. Various animal models have been developed to investigate the behavioural consequences of alterations in *RBFOX1*. Both zebrafish and mouse models with *rbfox1* mutations exhibit behavioural alterations consistent with the symptomatology of *RBFOX1*-associated disorders [5,7,11]. In the case of zebrafish, *rbfox1* mutant lines (*rbfox1^sa15940^* and *rbfox1^del19^*) have been studied for behavioural alterations. The *rbfox1* mutant line *rbfox1^sa15940^* used in this paper has a point mutation leading to the in-frame skipping of the second exon (or third in isoform rbfox1-206), resulting in the expression of a shorter, aberrant mRNA, whereas the *rbfox1^del19^* line has a deletion of 19 bp, which is also located in the second exon, leading to a frameshift mutation and a premature stop codon [11]. Both lines exhibit hyperactivity and alterations in social behaviour, specifically social novelty preference and/or shoaling behaviour [11]. Additionally, *rbfox1^sa15940^* mutants display altered aggressive behaviour [11]. *Rbfox1* knockout mice also demonstrate hyperactivity and altered aggressive and social interaction behaviour, along with stereotyped behaviour and impairments in fear acquisition and fear extinction [7].

Zebrafish serve not only as a valuable tool for studying the contributions and mechanisms of genes in complex psychiatric disorders but also for exploring potential pharmacological treatments. The substantial homology of receptors and enzymes between zebrafish and humans allows for comparable influences of pharmacological interventions, rendering them a time- and cost-efficient model for medical research [12,13,14]. Selective serotonin reuptake inhibitors (SSRIs), such as fluoxetine, are among the recommended pharmacological treatments for various *RBFOX1*-associated disorders, including MDD, OCD, and anxiety, making them one of the most commonly prescribed classes of medication worldwide for these disorders [14,15,16,17,18]. Fluoxetine has been studied extensively in zebrafish, both with regard to its clinical applications and its potential aquatic toxicity [14,19,20,21,22]. As an SSRI, its primary mechanism involves binding to the serotonin (5-hydroxytryptamine, 5-HT) transporter on the presynaptic membrane, thereby preventing 5-HT reuptake. This process elevates 5-HT concentrations in the synaptic cleft, prolonging the activation of postsynaptic receptors [14].

In zebrafish, 5-HT is involved in regulating fear, anxiety, and stress-related behaviours, exhibiting noteworthy variations among zebrafish strains [22]. Additionally, aggressive behaviour and locomotion are influenced by 5-HT, where higher levels are associated with both decreased locomotion and reduced aggression [23,24]. Considering the role of 5-HT in locomotion and aggressive behaviour, we hypothesise that alterations in serotonergic neurotransmission could be implicated in the observed behavioural changes in *rbfox1^sa15940/sa15940^* zebrafish [11]. However, the effects of *rbfox1* on serotonergic changes and the subsequent behavioural alterations have been largely unexplored. In this study, we investigate the contribution of 5-HT neurotransmission to the *rbfox1^sa15940/sa15940^* phenotype in adult zebrafish by characterising neurotransmitter levels in the brain and examining the effects of the SSRI fluoxetine on behaviour.

## 2. Results

### 2.1. Neurotransmitter Alterations in rbfox1^sa15940/sa15940^ Zebrafish

**Figure 1:**
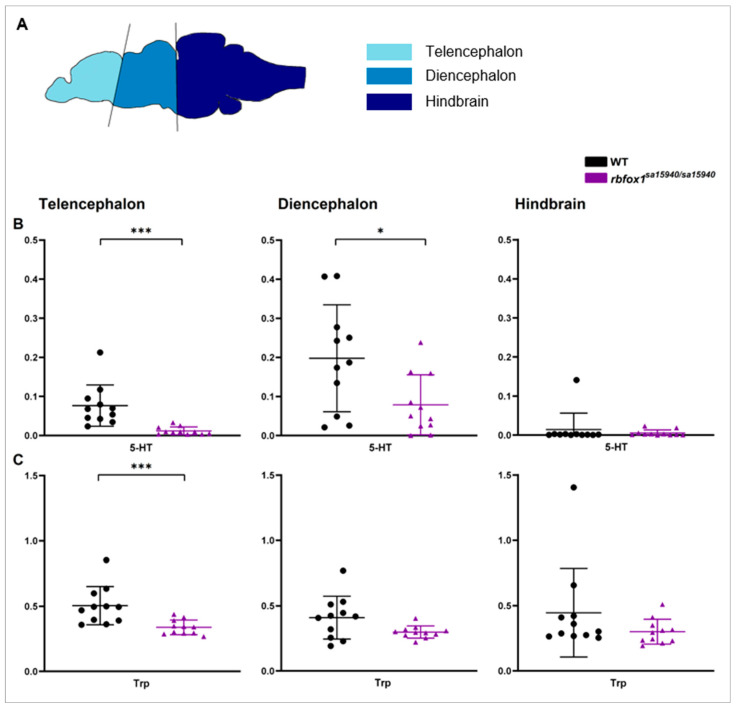
**Basal serotonin and tryptophan levels in wildtype and *rbfox1^sa15940/sa15940^* zebrafish**. (**B**) Serotonin (5-HT) and (**C**) Tryptophan (Trp) levels of adult zebrafish were measured via ultra-high-performance liquid chromatography–tandem mass spectrometry (UPLC/MS-MS) in the telencephalon, diencephalon, and hindbrain (**A**) and normalised to phenylalanine concentration of the respective sample; WT = wildtype, *rbfox1^sa15940/sa15940^* = homozygous mutants, 5-HT = Serotonin, Trp = Tryptophan; N = 11 per group; two-tailed Mann–Whitney test; mean ± SD; * p_adj_ < 0.05, *** p_adj_ < 0.001.

Basal serotonin (5-HT) and tryptophan (Trp) levels were assessed in *rbfox1^sa15940/sa15940^* and WT adult zebrafish across three distinct brain regions: telencephalon, diencephalon, and hindbrain (Figure 1A). Phenylalanine (Phe) levels served as a normalisation reference. Prior to normalisation to Phe, 5-HT levels in *rbfox1^sa15940/sa15940^* zebrafish were significantly lower than in WT zebrafish in the telencephalon and diencephalon (p_adj_ < 0.0001 and p_adj_ = 0.0336, respectively; 5-HT mean ± SD WT: 0.1140 ± 0.0646, *rbfox1^sa15940/sa15940^*: 0.0300 ± 0.0283 and 5-HT mean ± SD WT: 0.4297 ± 0.1560, *rbfox1^sa15940/sa15940^*: 0.2571 ± 0.1765, respectively) but not the hindbrain (p_adj_ = 0.3316; 5-HT mean ± SD WT: 0.0324 ± 0.0852, *rbfox1^sa15940/sa15940^*: 0.0240 ± 0.0292) (Supplementary Figure S1A). Both Trp and Phe levels did not show significant differences in any region, except for Phe levels in the telencephalon, which were slightly higher in *rbfox1^sa15940/sa15940^* zebrafish (p_adj_ = 0.0104; telencephalon: Phe mean ± SD WT: 1.8180 ± 1.1610, *rbfox1^sa15940/sa15940^*: 2.5170 ± 0.5780 and Trp mean ± SD WT: 0.8592 ± 0.5599, *rbfox1^sa15940/sa15940^*: 0.8255 ± 0.1139; diencephalon: Phe mean ± SD WT: 3.6900 ± 2.7970, *rbfox1^sa15940/sa15940^*: 4.3300 ± 1.7290 and Trp mean ± SD WT: 1.1610 ± 0.4104, *rbfox1^sa15940/sa15940^*: 1.2110 ± 0.8592; hindbrain: Phe mean ± SD WT: 5.4290 ± 2.7690, *rbfox1^sa15940/sa15940^*: 5.4880 ± 1.5500 and Trp mean ± SD WT: 1.7530 ± 0.4270, *rbfox1^sa15940/sa15940^*: 1.5880 ± 0.2946) (Supplementary Figure S1B,C). After normalisation to Phe, both 5-HT and Trp concentrations were significantly lower in *rbfox1^sa15940/sa15940^* zebrafish compared to WT fish in the telencephalon (p_adj_ < 0.0001 and p_adj_ = 0.0006, respectively; 5-HT mean ± SD WT: 0.0764 ± 0.0528, *rbfox1^sa15940/sa15940^*: 0.0118 ± 0.0100, Trp mean ± SD WT: 0.5045 ± 0.1467, *rbfox1^sa15940/sa15940^*: 0.3384 ± 0.0562) (Figure 1B,C). In the diencephalon, basal 5-HT levels were significantly lower in *rbfox1^sa15940/sa15940^* zebrafish than in WT fish (p_adj_ = 0.0336; 5-HT mean ± SD WT: 0.1978 ± 0.1368, *rbfox1^sa15940/sa15940^*: 0.0787 ± 0.0768), while Trp levels did not show a significant difference between the groups (p_adj_ = 0.0652, Trp mean ± SD WT: 0.4090 ± 0.1636, *rbfox1^sa15940/sa15940^*: 0.2985 ± 0.0466). In the hindbrain, no differences were detected between the two genotypes in 5-HT or Trp levels (p_adj_ = 0.3316 and p_adj_ = 0.1932, respectively; 5-HT mean ± SD WT: 0.0142 ± 0.0421, *rbfox1^sa15940/sa15940^*: 0.0055 ± 0.0078, Trp mean ± SD WT: 0.4455 ± 0.3392, *rbfox1^sa15940/sa15940^*: 0.3007 ± 0.0951).

### 2.2. Partial Reversal of Behavioural Alterations in rbfox1^sa15940/sa15940^ Zebrafish with Fluoxetine

Given the significantly lower basal levels of 5-HT in the telencephalon and diencephalon of *rbfox1^sa15940/sa15940^* zebrafish compared to WT fish, we hypothesised that acute exposure to an SSRI like fluoxetine could potentially rescue some of the previously observed behavioural alterations in the *rbfox1^sa15940/sa15940^* line, including hyperactivity, aggressive, and altered social behaviour [11].

#### 2.2.1. Open Field

**Figure 2:**
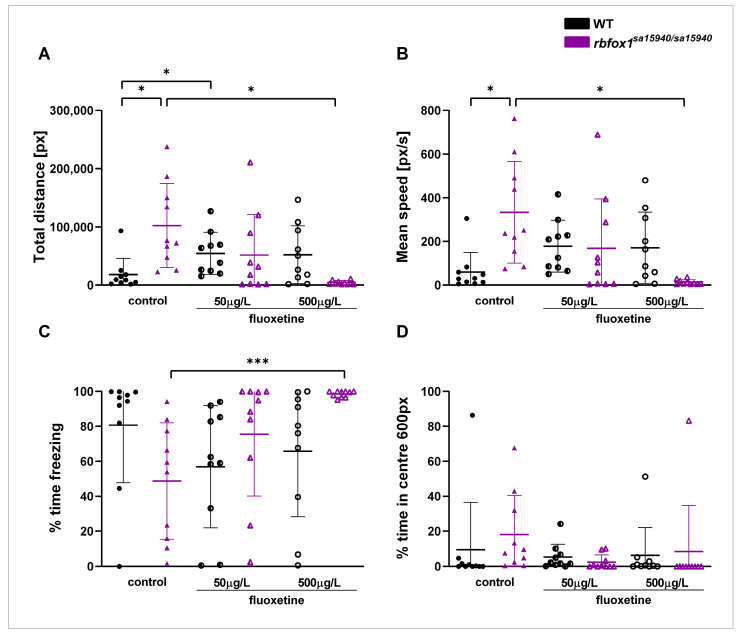
**Behaviour of wildtype and *rbfox1^sa15940/sa15940^* zebrafish after exposure to fluoxetine in the Open Field.** Adult zebrafish were tested in the Open Field paradigm after exposure to water (control group) or to different concentrations of fluoxetine for one hour prior to testing. (**A**) Total swimming distance; (**B**) mean speed; (**C**) time spent freezing; (**D**) time spent in the inner 50% of the arena; WT = wildtype, *rbfox1^sa15940/sa15940^* = homozygous mutants, control = 0 µg/L fluoxetine, N = 10 per group; permutation two-way ANOVA with interaction followed by *post hoc* permutational analysis using Benjamini and Hochberg correction; mean ± SD; * p_adj_ < 0.05, *** p_adj_ < 0.001.

In the Open Field paradigm, *rbfox1^sa15940/sa15940^* zebrafish exhibited behavioural alterations consistent with prior findings, displaying pronounced hyperactivity [11]. Adult *rbfox1^sa15940/sa15940^* and WT zebrafish of mixed sexes were exposed to either 0 µg/L (control group), 50 µg/L (C1), or 500µg/L (C2) fluoxetine for one hour before being tested in the Open Field paradigm. Hyperactivity observed in *rbfox1^sa15940/sa15940^* zebrafish could be reversed by acute exposure to the higher of the two tested fluoxetine concentrations (C2, 500 µg/L) for one hour (Figure 2).

The total swimming distance was significantly higher in the *rbfox1^sa15940/sa15940^* control group than in the WT control group (p_adj_ = 0.0126). Zebrafish in the *rbfox1^sa15940/sa15940^* C2 group had a significantly lower total swimming distance than the *rbfox1^sa15940/sa15940^* control group (p_adj_ = 0.0126), with no significant difference from the WT control group (p_adj_ = 0.1058). The swimming distance in the WT C1 group was significantly higher than in the WT control group (*p* = 0.0492).

Swimming speed in the *rbfox1^sa15940/sa15940^* control group was significantly higher than in the WT control group (p_adj_ = 0.0116), whereas zebrafish in the *rbfox1^sa15940/sa15940^* C2 group had a significantly lower swimming speed than the *rbfox1^sa15940/sa15940^* control group (p_adj_ = 0.0116).

Fish in the *rbfox1^sa15940/sa15940^* group exposed to C2 of fluoxetine spent significantly more time freezing than the *rbfox1^sa15940/sa15940^* control group (p_adj_ = 0.0140), but no more than the WT control group (p_adj_ = 0.4120). There was no notable difference in freezing behaviour between the *rbfox1^sa15940/sa15940^* control group and the WT controls (p_adj_ = 0.1718). There were no significant differences detected in the time spent in the centre of the arena between the groups.

Acute exposure to either of the fluoxetine concentrations did not significantly alter the behaviour of the WT fish apart from the total distance measure in the WT C1 group. In the tested behaviours, no significant differences were found between the *rbfox1^sa15940/sa15940^* zebrafish exposed to C1 of fluoxetine and the WT or *rbfox1^sa15940/sa15940^* control groups.

Consequently, we used only the C2 fluoxetine concentration (500 µg/L) for subsequent tests.

#### 2.2.2. Aggressive Behaviour of *rbfox1^sa15940/sa15940^* Zebrafish under the Influence of Fluoxetine

**Figure 3:**
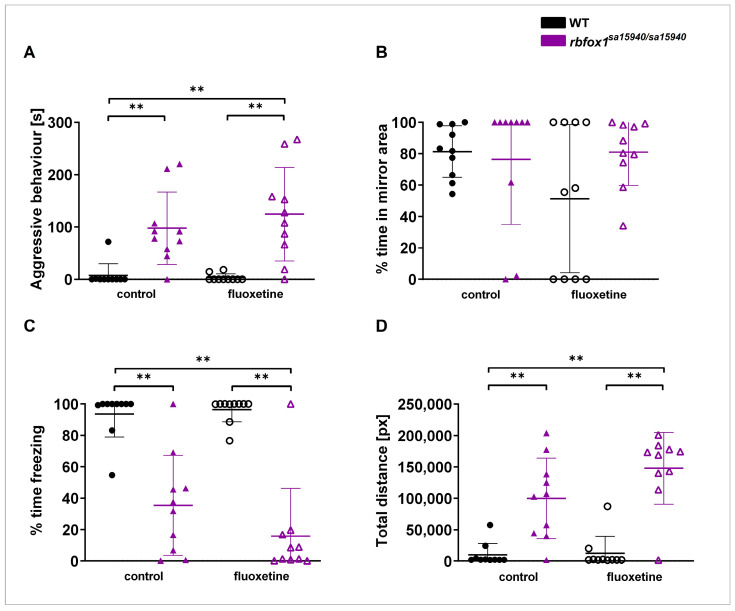
**Aggressive behaviour in wildtype and *rbfox1^sa15940/sa15940^* zebrafish after exposure to 500 µg/L fluoxetine**. Zebrafish were tested in the Mirror test to assess aggressive behaviour after exposure to water (control group) or to 500 µg/L fluoxetine for one hour prior to testing. (**A**) Time displaying aggressive behaviour; (**B**) time spent in 25% of the arena close to the mirror; (**C**) time spent freezing; (**D**) total swimming distance; WT = wildtype, *rbfox1^sa15940/sa15940^* = homozygous mutants, control = 0 µg/L fluoxetine, fluoxetine = 500 µg/L fluoxetine; N = 10 per group; permutation two-way ANOVA with interaction followed by *post hoc* permutational analysis using Benjamini and Hochberg correction; mean ± SD; ** p_adj_ < 0.01.

The Mirror test serves as a paradigm for measuring aggressive behaviour (Figure 3). Here, the *rbfox1^sa15940/sa15940^* control group and the *rbfox1^sa15940/sa15940^* fluoxetine group exhibited significantly more aggressive behaviour than the WT control group (p_adj_ = 0.0038 and p_adj_ = 0.0038, respectively). The *rbfox1^sa15940/sa15940^* fluoxetine group exhibited significantly more aggressive behaviour than the WT fluoxetine group (p_adj_ = 0.0038). There was no significant difference in the time spent in the 25% of the arena close to the mirror between groups. Both the *rbfox1^sa15940/sa15940^* control group and the *rbfox1^sa15940/sa15940^* fluoxetine group spent significantly less time freezing than the WT control group (p_adj_ = 0.0035 and p_adj_ = 0.0035, respectively). The *rbfox1^sa15940/sa15940^* fluoxetine group spent significantly less time freezing than the WT fluoxetine group (p_adj_ = 0.0035). Furthermore, the *rbfox1^sa15940/sa15940^* control group and the *rbfox1^sa15940/sa15940^* fluoxetine group swam a significantly longer distance than the WT control group (p_adj_ = 0.0040 and p_adj_ = 0.0040, respectively). The *rbfox1^sa15940/sa15940^* fluoxetine group swam a significantly longer distance than the WT fluoxetine group (p_adj_ = 0.0035).

#### 2.2.3. Social Behaviour of *rbfox1^sa15940/sa15940^* Zebrafish under the Influence of Fluoxetine

In the Open Field paradigm, exposure to 500 µg/L of fluoxetine was found to reverse some of the behavioural alterations observed in the *rbfox1^sa15940/sa15940^* zebrafish. We, therefore, sought to determine if these effects extended to paradigms for social behaviour, including the VMSP paradigm in which we had not observed previously differences in this *rbfox1^sa15940^* mutant line [11].

##### Social Novelty Preference Test

**Figure 4:**
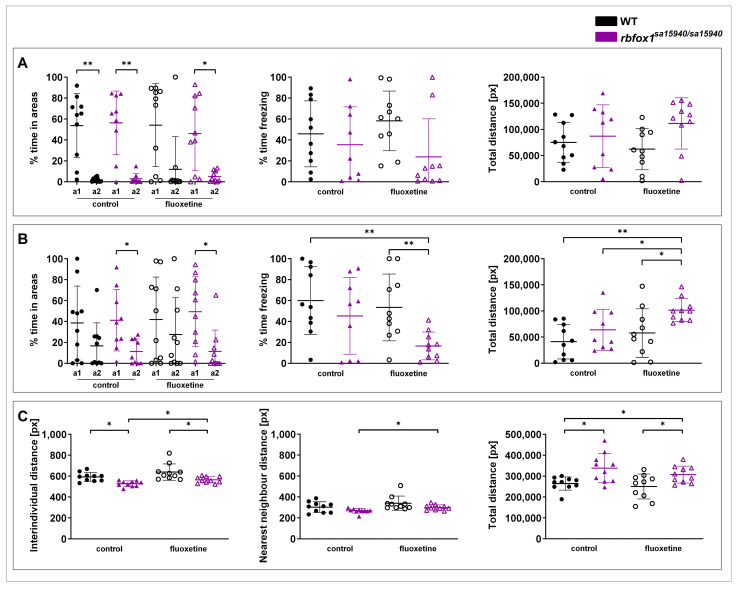
**Social behaviour in wildtype and *rbfox1^sa15940/sa15940^* zebrafish after exposure to 500 µg/L fluoxetine.** Zebrafish were tested in the Visually Mediated Social Preference (VMSP) and the Shoaling paradigm to assess social behaviour after being exposed to water (control group) or to 500 µg/L fluoxetine for one hour prior to testing. (**A**) VMSP part 1 time spent in the zone close to the first group of strangers (a1) versus the opposite area (a2), time spent freezing, and total swimming distance; (**B**) VMSP part 2 time spent in the zone close to the first group of strangers (a1) versus the area close to the second group of strangers (a2), time spent freezing, and total swimming distance; (**C**) shoaling interindividual distance, nearest neighbour distance, and total swimming distance; WT = wildtype, *rbfox1^sa15940/sa15940^* = homozygous mutants, control = 0 µg/L fluoxetine, fluoxetine = 500 µg/L fluoxetine; N = 10 per group for all groups except the *rbfox1^sa15940/sa15940^* control group in the VMSP (N = 9); permutation two-way ANOVA with interaction followed by *post hoc* permutational analysis using Benjamini and Hochberg correction except for % time in areas: Wilcoxon matched-pairs signed rank test; mean ± SD; * p/p_adj_ < 0.05, ** p/p_adj_ < 0.01.

The Visually Mediated Social Preference (VMSP) paradigm comprises two parts. Part 1 assesses social preference, measuring whether the tested fish prefers to swim closer to a group of three unfamiliar zebrafish (strangers 1) (Figure 4A). In part 2, the social novelty preference step, another group of three novel zebrafish (strangers 2) is introduced, and it is measured whether the tested fish prefers to swim closer to the first or second group (Figure 4B). In part 1, no significant differences were found between the tested groups regarding the time spent freezing and the total distance. Both the WT and the *rbfox1^sa15940/sa15940^* control group, as well as the *rbfox1^sa15940/sa15940^* fish exposed to fluoxetine, spent significantly more time close to strangers 1 than the opposite area (*p* = 0.0020, *p* = 0.0078, and *p* = 0.0117, respectively). Although not significant, WT zebrafish exposed to fluoxetine displayed a similar tendency to spend more time next to strangers 1 (p_adj_ = 0.1309). In part 2, neither the WT control group nor the WT fish exposed to fluoxetine exhibited a clear preference for either group of strangers. In contrast, both the *rbfox1^sa15940/sa15940^* control group and the *rbfox1^sa15940/sa15940^* fluoxetine group demonstrated a significant preference for the group of strangers 1 (*p* = 0.0195 and *p* = 0.0371, respectively). Additionally, the *rbfox1^sa15940/sa15940^* fluoxetine group spent significantly less time freezing than the WT control and WT fluoxetine groups (p_adj_ = 0.0065 and p_adj_ = 0.0070, respectively). The *rbfox1^sa15940/sa15940^* fluoxetine group also swam a significantly longer distance than the WT control, the *rbfox1^sa15940/sa15940^* control, and the WT fluoxetine groups (p_adj_ = 0.0010, p_adj_ = 0.0298, and p_adj_ = 0.0298, respectively).

##### Shoaling

The Shoaling paradigm measures the behaviour of zebrafish in a group (Figure 4C). Here, the *rbfox1^sa15940/sa15940^* control zebrafish had a slightly lower interindividual distance (IID) than the WT control group (p_adj_ = 0.0118), a difference not observed in the *rbfox1^sa15940/sa15940^* fluoxetine group (p_adj_ = 0.1193).

The *rbfox1^sa15940/sa15940^* fluoxetine group had a slightly higher IID than the *rbfox1^sa15940/sa15940^* control group (p_adj_ = 0.0222) and a slightly lower IID than the WT fluoxetine group (p_adj_ = 0.0118).

The *rbfox1^sa15940/sa15940^* fluoxetine group had a slightly higher nearest neighbour distance (NND) than the *rbfox1^sa15940/sa15940^* control group (p_adj_ = 0.0400), with no significant difference to the WT control group (p_adj_ = 0.8470).

Both the *rbfox1^sa15940/sa15940^* control and the *rbfox1^sa15940/sa15940^* fluoxetine group swam a significantly longer distance than the WT control group (p_adj_ = 0.0375 and p_adj_ = 0.0417, respectively). The total distance of the *rbfox1^sa15940/sa15940^* fluoxetine group was significantly higher than the total distance of the WT fluoxetine group (p_adj_ = 0.0417).

Consistent with our observations in the Open Field paradigm, acute exposure to fluoxetine did not significantly alter the behaviour of the WT fish in any of the tested social paradigms with respect to time spent freezing, total swimming distance, IID, or NND.

## 3. Discussion

*RBFOX1* has been linked to numerous neurodevelopmental and psychiatric disorders, whose treatment usually includes the commonly prescribed SSRI fluoxetine [1,3,8,11,25,26]. This is the first study investigating the link between this gene and serotonergic neurotransmission. In this study, we examined the impact of a *rbfox1* mutation on 5-HT levels in adult zebrafish and explored the extent to which observed behavioural alterations in our *rbfox1^sa15940/sa15940^* model can be attributed to serotonergic functions. To achieve this, we measured levels of 5-HT and its precursor tryptophan in the brains of adult zebrafish, revealing significantly lower 5-HT concentrations in the telencephalon and diencephalon, along with lower concentrations of tryptophan in the telencephalon in *rbfox1^sa15940/sa15940^* compared to WT fish. Subsequently, we investigated the effect of the SSRI fluoxetine and observed a partial reversal of the hyperactivity and altered shoaling behaviour of the *rbfox1^sa15940/sa15940^* zebrafish when acutely treated with fluoxetine. Our study suggests that alterations in 5-HT neurotransmission contribute, to a certain extent, to the phenotype observed in *rbfox1* mutant zebrafish.

### 3.1. Serotonin Imbalances Associated with the rbfox1^sa15940^ Mutation

In our UPLC/MS-MS analysis, we found reduced levels of 5-HT in the telencephalon and diencephalon, as well as reduced levels of tryptophan in the telencephalon in *rbfox1^sa15940/sa15940^* compared to WT fish. In adult zebrafish, serotonergic nuclei are primarily located in the diencephalon and hindbrain. Unlike mammals, zebrafish also have serotonergic nuclei along the spinal cord [23,27,28]. Serotonergic nuclei, as described by Lillesaar et al. (2011), are located in the diencephalon within the pineal gland, the boundary region of the thalamus and pretectum, the posterior tuberculum, and the hypothalamus [23]. Additionally, serotonergic nuclei in the hindbrain are found in the superior and inferior raphe populations, the reticular formation of the Medulla oblongata, and the area postrema [23]. These nuclei project primarily to different fore- and midbrain areas, with only a few projections reaching some hindbrain regions [23,27]. The neuronal terminals of the projecting neurons are primarily located in the forebrain and diencephalon, which could explain the decreased 5-HT levels in the telencephalon and diencephalon in *rbfox1* mutant zebrafish, but not the hindbrain [23,27,28]. The alterations in the 5-HT levels may have various explanations. One possibility is impaired 5-HT synthesis; we observed reduced basal levels of the 5-HT precursor tryptophan in the telencephalon of *rbfox1^sa15940/sa15940^* zebrafish suggesting insufficient availability of tryptophan, which, in turn, could impede 5-HT synthesis. However, as the observed differences in Trp levels only become significant after normalisation to Phe, effects other than the availability of Trp could be the true reason for the lowered 5-HT levels.

### 3.2. Influence of Fluoxetine on Zebrafish Behaviour

In zebrafish, 5-HT is involved in modulating various behaviours, including locomotion, stress, fear, anxiety, appetite, aggression, and social behaviour [14,23]. For this study, we selected the Open Field, the VMSP, the Shoaling, and the Mirror tests, as they have been used in previous investigations of two lines of *rbfox1* mutant zebrafish [11]. Since neither of the two *rbfox1* mutant zebrafish lines used in the aforementioned study by Antón-Galindo et al. (2024) exhibited increased anxiety behaviour in the Black and White test, we omitted this test from the present study. Given the absence of a standardised protocol for testing fluoxetine in zebrafish, we initially examined two different concentrations in the Open Field paradigm [29,30]. We observed an effect only at the higher of the two doses (500 µg/L), which was subsequently chosen for the remaining experiments.

The Open Field paradigm assesses locomotion and thigmotaxic behaviour in a novel environment. We noted pronounced freezing behaviour in WT fish, consistent with prior findings from this strain [11]. The *rbfox1^sa15940/sa15940^* control group displayed hyperactive behaviour, as previously observed, potentially resulting from the deficiency in 5-HT [11,23,24]. Fluoxetine reduced hyperactive behaviour and increased freezing in *rbfox1^sa15940/sa15940^* zebrafish, prompting them to exhibit behaviour more akin to WT fish. Exposing WT fish to 50 µg/L fluoxetine led to a significant increase in locomotion; however, this effect was not significant in the WT group exposed to 500 µg/L fluoxetine. Similarly to the Open Field test, the *rbfox1^sa15940/sa15940^* control group displayed hyperactive behaviour in the Shoaling test, which was slightly decreased in the *rbfox1^sa15940/sa15940^* fluoxetine group. In contrast to the Open Field test, this hyperactive behaviour was not fully rescued by fluoxetine. Reduced swimming activity in zebrafish exposed to fluoxetine has been reported before, however, these results are not consistent across studies [14]. Interestingly, one study found that 5-HT that is released from the brainstem and spinal cord reduced fictive locomotion in adult zebrafish [24]. An increase in 5-HT concentration induced by fluoxetine in the brains of the *rbfox1^sa15940/sa15940^* zebrafish could therefore directly impact their locomotion, diminish hyperactive behaviour, and contribute to restoring a wildtype-like phenotype in these paradigms. As the WT fish in this study exhibited strong freezing behaviour under control conditions and after exposure to fluoxetine, it remains unclear whether fluoxetine would lead to a decrease in locomotion in the WT fish.

In contrast to the Open Field and Shoaling tests, exposure to fluoxetine during the second part of the VMSP and the aggression test did not result in a decrease in the total swimming distance in *rbfox1^sa15940/sa15940^* zebrafish compared to the WT control group. Furthermore, there was no observable increase in freezing in the *rbfox1^sa15940/sa15940^* fluoxetine group. This discrepancy may be attributed to distinct extrinsic and intrinsic motivations influencing locomotor behaviour in the different tests. The Open Field exposes a fish to a novel arena devoid of external stimulations, while in the Shoaling test, the tested group of fish is familiar with each other as they have been housed in the same tank. However, in the VMSP and the Mirror test, the tested fish encounters groups of strangers and does not recognise itself in the mirror, respectively. The sight of unfamiliar peers may agitate the fish, leading to hyperactive behaviour. In these tests, intrinsic freezing behaviour might be concealed by a general state of arousal induced by the external stimulus of encountering unfamiliar fish, as opposed to the controlled conditions of the Open Field and Shoaling tests [31]. Another possible explanation could be the involvement of additional neurotransmitters in the behaviour observed in the VMSP and Mirror tests, which may not be the primary targets of the SSRI fluoxetine.

In the first part of the VMSP test, WT zebrafish exposed to fluoxetine spent slightly less time with the group of unfamiliar fish. This could be a consequence of decreased social interest following acute exposure to fluoxetine, as demonstrated previously [14]. However, the observed effect in this study was relatively small and may be a statistical artefact.

In the social novelty preference step of the VMSP test, *rbfox1^sa15940/sa15940^* zebrafish of both the control and the fluoxetine group displayed a slight decrease in social novelty preference, as they spent significantly more time close to the first group of unfamiliar fish. In the Shoaling test, the *rbfox1^sa15940/sa15940^* control group had a significantly lowered interindividual distance, which was rescued in the *rbfox1^sa15940/sa15940^* fluoxetine group. The nearest neighbour distance was also slightly increased in the *rbfox1^sa15940/sa15940^* fluoxetine group compared to the *rbfox1^sa15940/sa15940^* control group. This could indicate that fluoxetine helps to re-establish a WT-like phenotype in the *rbfox1^sa15940/sa15940^* fish in these paradigms. Considering the lack of preference for the group of unfamiliar fish in part 1 of the VMSP in the WT fish that were exposed to fluoxetine, these findings could also indicate that fluoxetine lowered the social interest in these groups. However, the observed effects are quite small.

In the aggression test, *rbfox1^sa15940/sa15940^* zebrafish displayed increased aggressive behaviour both in the control condition and after exposure to fluoxetine. *RBFOX1* has been identified as a strong candidate gene for aggression in human and rodent studies, both directly and through the regulation of the expression of other genes associated with aggression [5,11,25]. *rbfox1^sa15940/sa15940^* zebrafish from this line have previously shown increased aggression [11]. One possible explanation for increased aggression in *rbfox1^sa15940/sa15940^* zebrafish could lie in their altered 5-HT levels. Interestingly, innately high levels of 5-HT and acute exposure to fluoxetine have been linked to reduced aggressive behaviour in wildtype fish [21,23]. We found 5-HT levels to be reduced in the *rbfox1^sa15940/sa15940^* zebrafish, which could be a reason for their increased aggression. However, the exposure to fluoxetine did not lower the displayed aggressive behaviour in the tested *rbfox1^sa15940/sa15940^* zebrafish. This could stem from various reasons. Although serotonergic functions have been linked to aggression, the aggressive behaviour in *rbfox1^sa15940/sa15940^* zebrafish may involve other underlying neurological mechanisms, possibly interacting with the serotonergic system, which conceals the effects of fluoxetine on the aggressive behaviour. For instance, the dopaminergic system, in conjunction with the 5-HT system, has been implicated in zebrafish aggression in the Mirror test and in the dyadic interaction of two fish, while in rats and humans, the interaction of the dopaminergic and noradrenergic systems plays a role in aggressive behaviour [32]. Given that *RBFOX1* exerts pleiotropic effects on numerous genes, it is plausible that other *rbfox1* targets outside the serotonergic system, and therefore largely unaffected by the acute fluoxetine exposure, are involved in the aggressive behaviour [5,25]. As both the WT control group and the WT group exposed to fluoxetine spent most of the time freezing, the potential effects of fluoxetine on their behaviour could not be discerned. It is important to note that findings about altered aggressive behaviour differ among *rbfox1* animal models: The *rbfox1^sa15940^* line used in this study has displayed increased aggression before; however, a *rbfox1* mutant zebrafish line with a TU background did not exhibit altered aggressive behaviour, and male mice in a *Rbfox1* knockdown model even showed decreased aggression [7,11]. This underscores the influence of the genetic background, indicating varied genetic interactions between *rbfox1* and other aggression-related genes. Furthermore, while both zebrafish and *rbfox1* mutant mice were tested for aggressive behaviour, the tests themselves might not be directly comparable. Zebrafish were subjected to the Mirror test, assessing aggressive behaviour toward a perceived stranger in a novel environment, while mice were tested in a resident-intruder test [7]. Therefore, the underlying motivation for aggressive behaviour may vary between these paradigms.

Our study suggests that behavioural changes in *rbfox1^sa15940/sa15940^* zebrafish may also be influenced by mechanisms other than serotonergic neurotransmission, as not all the phenotypic alterations in mutant zebrafish could be reversed upon exposure to fluoxetine. Further studies should explore the involvement of neurotransmission systems beyond the serotonergic pathway.

### 3.3. Limitations of This Study

A limitation of this study is the inability to weigh the extracted brain areas for the UPLC/MS-MS analysis to normalise the neurotransmitters to tissue weight. Additionally, normalisation to total protein content was not feasible since protein measures were low and not reliable. To address this, we normalised the neurotransmitter levels to the corresponding Phe concentration, a method employed previously [33]. In our samples, Phe exhibited no differences between genotypes, maintaining similar levels across regions (proportional to their sizes). Only a slight increase in Phe levels was observed in telencephalon of *rbfox1^sa15940/sa15940^*. Even prior to normalisation to Phe, 5-HT levels were significantly lower in *rbfox1^sa15940/sa15940^* zebrafish compared to WT in the telencephalon and diencephalon, indicating robustness of the observed differences in 5-HT between genotypes.

We quantified 5-HT and Trp levels in 3-month-old zebrafish, and behaviour was assessed in 4–5-month-old fish. Although we do not expect differences in adults since there are no significant changes in maturation during this period, we cannot discard them. Furthermore, we were not able to analyse the levels of 5-HT and Trp in the brains of the zebrafish after exposure to fluoxetine and behavioural testing, as, for technical reasons, the brains could not be obtained under the same conditions as the brains of the naïve fish.

Generally, there appears to be a pronounced difference between acute and chronic exposure to fluoxetine, with the latter yielding more consistent and anxiolytic effects [19,30,31]. A limitation of our study lies in that we could only test acute exposure to fluoxetine. Acutely, fluoxetine operates by blocking the serotonin transporter (SERT), thereby limiting the 5-HT reuptake from the synaptic cleft and increasing 5-HT concentrations in the brain [14,19,26,31]. It is possible that in the tested doses of fluoxetine in this study, its acute effects are only measurable in the *rbfox1^sa15940/sa15940^* zebrafish, given their lower base levels of 5-HT in the telencephalon and diencephalon. Additionally, unlike many studies on fluoxetine in zebrafish that induce acute stress before testing, our study examined the impact of fluoxetine on the “baseline behaviour” of WT and *rbfox1^sa15940/sa15940^* zebrafish. This difference in approach may explain the lack of similar effects on WT fish compared to other groups. Furthermore, our sample size might have been insufficient to uncover all the effects of the *rbfox1* mutation and/or fluoxetine on the tested behaviours. Adhering to the “three R principle”, we selected the smallest possible sample size based on previous studies, which introduces a potential limitation.

Pooling results from male and female zebrafish is another potential limitation. Many of the psychiatric conditions associated with *RBFOX1* exhibit different prevalence in men and women, possibly involving distinct mechanisms. However, as previous analyses did not reveal behavioural differences between the sexes in the employed paradigms, both male and female zebrafish were tested, and the results were pooled [11]. Thirty to 70% of the tested zebrafish appeared to be female, although it is important to note that the determination of zebrafish sex was based on the morphological assessment by the experimenter, which may be prone to errors due to the visual similarity of male and female zebrafish at this age.

Finally, we did not explore the mechanisms by which the *rbfox1* mutation presented in this study alters serotonin neurotransmission and behaviour. This should be investigated and clarified in future studies.

## 4. Materials and Methods

### 4.1. Zebrafish Lines and Husbandry

The zebrafish line employed for behavioural experiments, *rbfox1^sa15940^* on a Tübingen Long-fin background (TL), was obtained from the European Zebrafish Resource Center of the Karlsruhe Institute of Technology. As described previously [11], this line carries a point mutation at the −2 position of a 3′ acceptor splicing site of the *rbfox1* gene in the intron before the second or third exon of all annotated isoforms except one (A > T, Chr3:28068329, GRCz11), which results in an exon skipping that leads to a shorter aberrant *rbfox1* mRNA [11]. The expression of the other annotated *rbfox* genes in zebrafish is not affected by this mutation [11]. Homozygous *rbfox1* mutant zebrafish (*rbfox1 ^sa15940/sa15940^*) and wildtype fish (WT) of mixed sexes (estimated percentage of female fish between 30 and 70 per test) were used for all experiments. Zebrafish were maintained in tanks at 28.5 °C under a 14:10 h light–dark cycle following standard procedures. Ethical approval for all experiments was obtained from the Animal Welfare and Ethical Review Board of the Generalitat de Catalunya.

### 4.2. Quantification of Basal Serotonin and Tryptophan Levels

We quantified in the brain the levels of serotonin (5-HT) in the brain, its precursor tryptophan (Trp), and phenylalanine (Phe) used as a reference for normalisation, as previously reported [33]. For that, a total of 11 WT and 11 *rbfox1^sa15940/sa15940^* zebrafish were euthanised at 3 months of age through immersion in ice water. Their brains were extracted in a Petri dish containing 1X phosphate-buffered saline (PBS) solution using a Leica EZ4D microscope and separated into four areas: telencephalon, diencephalon, hindbrain (Figure 1A), and optic tectum; however, the latter was not used for the analysis. The telencephalon, diencephalon, and hindbrain samples were put into previously frozen 1.5 mL reaction tubes and immediately put into dry ice. Until analysis, they were stored at −80 °C. Samples were sent to Hospital de Joan de Déu to analyse the concentrations of 5-HT, the 5-HT precursor tryptophan, and the amino acid phenylalanine with ultra-high-performance liquid chromatography–tandem mass spectrometry (UPLC-MS/MS) [34].

The frozen pellets were resuspended in 80 µL of phosphate-buffered saline. Cells were lysed via sonication, using cycles of 10 s (40% amplitude, on ice), and frozen at −80 °C until analysis. After thawing, sample aliquots were vortexed with 25 µL of the internal standard solution and 150 µL of methanol/0.1% formic acid to precipitate the protein samples. They were subsequently centrifuged for 10 min at 10 °C, and amino acid levels were measured in the supernatants by UPLC-MS/MS, as previously described [34]. For 5-HT, 10 µL of the cell supernatants were mixed with 10 µL of the internal standard solution (dopamine D4 as internal standard; Sigma-Aldrich REF 73483). Then, samples were centrifuged at 600× *g* for 10 min at 10 °C. Chromatographic conditions; column: CORTECS C18 2.1 × 150, 1.6 µm; mobile phase: water/formic acid (99.5%) and acetonitrile/formic acid (0.5%). All samples were analysed using a Waters ACQUITY UPLC H-class coupled to a Waters Xevo TQD triple quadrupole mass spectrometer using positive electrospray ionisation in the multiple reaction monitoring modes. Together with phenylalanine, tryptophan was also analysed by the same method. The results were normalised to phenylalanine concentration, a reliable technique established by the group [33].

### 4.3. Drugs

The fluoxetine used in these experiments was solid fluoxetine hydrochloride (SIGMA-ALDRICH, F132-10MG). A fluoxetine stock solution of 10 mg/L in deionised water was previously prepared following adequate safety protocols. On the day of testing, this solution was diluted with water from the zebrafish system to a concentration of 50 µg/L and 500 µg/L fluoxetine for the Open Field experiment, or 500 µg/L for the Visually Mediated Social Preference, the Shoaling, and the Mirror test.

### 4.4. Behavioural Experiments

Zebrafish aged 4–5 months were tested in four paradigms as described by Antón-Galindo et al. (2024) [11], namely the Open Field, the Visually Mediated Social Preference (VMSP), the Shoaling, and the Mirror tests. Around one week prior to testing, fish were randomly assigned to testing groups based on genotype and kept in separate housing tanks, with the caretakers being blind to the experimental design. Tanks were maintained under uniform environmental and housing conditions. The sample size required for behavioural experiments was calculated using GPower 3.1 [35].

Before testing, fish were placed in 1 L habituation tanks in the testing room for 55–65 min, exposed to the respective fluoxetine concentration (50 µg/L and 500 µg/L for the Open Field experiment, or 500 µg/L fluoxetine for the Visually Mediated Social Preference, the Shoaling, and the Mirror test), or water from the zebrafish system for the control group. For the Open Field, VMSP, and Mirror test, two fish were placed together in habituation tanks, as isolation might cause stress to zebrafish [36]. For the Shoaling test, five fish were habituated in their corresponding testing group. The testing order was structured to alternate between control groups (WT and *rbfox1^sa15940/sa15940^* at 0 µg/L fluoxetine) and fish groups exposed to fluoxetine (WT and *rbfox1^sa15940/sa15940^* at 50 µg/L and at 500 µg/L fluoxetine for the Open Field, and only at 500 µg/L for the Shoaling test, VSMP, and Mirror test). To ensure that there were no traces of fluoxetine left in the arena when starting a new round of testing, the water in the arena was renewed before the first control group of each round of testing.

Fish were recorded during testing with a digital camera and Streampix7 software (Norpix, Single Camera version). Testing arenas were surrounded by white material to reduce distractions and reflection. After testing, fish were placed into a 3 L tank and euthanised in groups of 15–20 fish at a time via immersion in a 1000 ppm Lidocaine solution for >10 min. After the Open Field experiments, the fish were not euthanised using Lidocaine, but by immersion in ice water right after testing.

Tracking analysis of the behavioural experiments was conducted as described by Antón-Galindo et al. (2024) [11]. Tracking utilised idtrackerai scripts (https://gitlab.com/polavieja_lab/idtrackerai, downloaded on 4 January 2021) and the trajectorytools module in Python (https://github.com/fjhheras/trajectorytools accessed on 4 January 2021) [37]. In the Mirror test, the time displaying aggressive behaviour was stopped manually.

#### 4.4.1. Open Field

In the Open Field test, WT and *rbfox1^sa15940/sa15940^* zebrafish were tested under two fluoxetine concentrations (50 µg/L or 500 µg/L) and compared to a control group in water to establish the adequate concentration for the other three paradigms. Each fish was introduced into a circular arena (43 cm diameter, water depth ~6–7 cm) novel to them and recorded for five minutes [11]. Analysed parameters included swimming speed, total distance, time spent in the inner 50% of the arena, and time freezing. A total of 10 zebrafish per group were tested between 10:00 h and 14:00 h.

#### 4.4.2. Mirror Test

To assess aggressive behaviour, the Mirror test was employed, following Norton et al. (2011) [38]. WT and *rbfox1^sa15940/sa15940^* zebrafish were tested at either 0 µg/L (control) or 500 µg/L fluoxetine. The arena comprised a single white chamber (24 cm × 12 cm, ~10 cm water depth) with a transparent wall at one of the shorter ends. Attached to one of the corners of the transparent wall was a mirror angled at 22°, in which the tested fish could see but not recognise itself. Fish were recorded for five minutes, with analysed parameters including swimming speed, total distance, time freezing, time displaying aggressive behaviour, and time spent in the 25% of the arena close to the mirror. A total of 10 zebrafish per group were tested between 9:00 h and 18:00 h.

#### 4.4.3. Social Novelty Preference Test

In the VMSP test following Carreño Gutierrez et al. (2019) [39], WT and *rbfox1^sa15940/sa15940^* zebrafish were tested at either 0 µg/L (control) or 500 µg/L fluoxetine. The VMSP arena had five chambers separated by transparent walls, with the central chamber (20 cm × 14 cm, ~10 cm water depth) being flanked by two smaller neighbouring chambers (10 cm × 7 cm each, ~10 cm water depth) on opposite sides (described in Figure 3A,B of Carreño Gutierrez et al. (2019) [39]). Each fish was recorded in two consecutive rounds of testing: In the first round, the social preference step, three unfamiliar zebrafish (strangers 1) were placed in one of the corner chambers. The tested fish was placed in the central chamber and recorded for five minutes. Immediately afterwards, in the social novelty preference step, three more unfamiliar zebrafish (strangers 2) were placed in the corner diagonally opposing the chamber of the first group of fish, and the tested fish was recorded for another five minutes. Total distance, time freezing, time spent in the quadrant close to the group of strangers 1, and time spent in the diagonally opposite quadrant (part 1 of the experiment), which contained the group of strangers 2 in part 2 of the experiment, were analysed. A total of 10 zebrafish were tested between 9:00 h and 18:00 h, except for the *rbfox1^sa15940/sa15940^* control group, where only 9 zebrafish were tested.

#### 4.4.4. Shoaling

In the Shoaling test, WT and *rbfox1^sa15940/sa15940^* zebrafish were tested at either 0 µg/L (control) or 500 µg/L fluoxetine. Five zebrafish were placed in the same arena as the Open Field, and, after a five-minute habituation period, they were recorded for ten minutes [40]. Analysed parameters included swimming speed, total distance, nearest neighbour distance (NND), and inter-individual distance (IID). A total of 10 zebrafish were tested between 10:00 h and 14:00 h.

### 4.5. Statistical Methods and Analysis

In comparing the levels of 5-HT and tryptophan across brain areas, the relative concentrations of 5-HT and tryptophan (normalised to phenylalanine) were compared between WT and *rbfox1^sa15940/sa15940^* for each brain region. Two-tailed Mann–Whitney tests were conducted using GraphPad Prism (GraphPad Prism version 8.0.2 for Windows, GraphPad Software, San Diego, CA, USA, www.graphpad.com, downloaded on 23 February 2022).

For the behavioural experiments involving fluoxetine, statistical analyses were carried out using GraphPad Prism (GraphPad Prism version 8.0.2 for Windows, GraphPad Software, San Diego, CA, USA, www.graphpad.com), as well as RStudio (R version 4.3.1). For each analysis, a permutation two-way ANOVA with interaction followed by post hoc permutational analysis using Benjamini and Hochberg correction was performed, except for the comparison of the percentage of time spent in the areas in the VMSP. In this case, we performed a Wilcoxon matched-pairs signed rank test. Only significant differences after Benjamini and Hochberg correction were considered and indicated (p_adj_), except for the comparison of the percentage of time spent in the areas in the VMSP, where no correction for multiple testing was applied. In the Open Field test, *rbfox1^sa15940/sa15940^* and WT fish exposed to two fluoxetine concentrations (C1 = 50 µg/L and C2 = 500 µg/L) were compared to the WT control group. Additionally, *rbfox1^sa15940/sa15940^* zebrafish exposed to C1 and C2 of fluoxetine were compared to the *rbfox1^sa15940/sa15940^* control group. For the other tests, *rbfox1^sa15940/sa15940^* and WT fish exposed to 500 µg/L fluoxetine were compared to the WT control group, and *rbfox1^sa15940/sa15940^* zebrafish exposed to 500 µg/L fluoxetine were compared to the *rbfox1^sa15940/sa15940^* control group and the WT fluoxetine group. In the VMSP, the time spent in two areas of the arena containing unfamiliar fish was compared within each group.

## 5. Conclusions

In conclusion, our study underscores the important role of *RBFOX1* in psychiatric phenotypes, specifically hyperactivity and aggression, and emphasises the involvement of serotoninergic neurotransmission in these traits. We highlight the changes observed in 5-HT associated with *rbfox1*, providing partial insight into the behavioural changes in *rbfox1* mutant zebrafish. Additionally, we highlight the pharmacological rescue of hyperactive behaviour in specific contexts by targeting serotonin reuptake. Future research should explore the influence of *RBFOX1* on other neurotransmitters or targets to understand the behavioural changes not accounted for by the identified serotonergic imbalance.

## Data Availability

Data is contained within the article and the supplementary material.

## Supplementary Materials

The following supporting information can be downloaded at: https://www.mdpi.com/article/10.3390/ph17020254/s1, Supplementary Figure S1: Raw levels of serotonin, tryptophan, and phenylalanine in wildtype and *rbfox1^sa15940/sa15940^* zebrafish.
