# Supplementary material for: Decreased Brain Serotonin in rbfox1 Mutant Zebrafish and Partial Reversion of Behavioural Alterations by the SSRI Fluoxetine
Source: Pharmaceuticals (Basel). 2024 Feb 16;17(2):254. doi: 10.3390/ph17020254 (PMC10891829; doi:10.3390/ph17020254)
Supplement: Supplementary file 1 [file pharmaceuticals-17-00254-s001.zip › pharmaceuticals-2748439-supplementary.pdf]

pharmaceuticals

Supplementary Material

Quantification of basal serotonin, tryptophan, and phenylalanine levels

Raw levels of serotonin (5-HT), tryptophan (Trp) and phenylalanine (Phe) were measured via ultra-high-performance liquid chromatography-tandem mass spectrometry (UPLC/MS-MS) as described in **4. Materials and Methods**. Without normalization, the 5-HT levels in *rbfox1*$^{sa15940/sa15940}$ zebrafish are significantly lower than in WT zebrafish in the telencephalon and diencephalon ($p_{adj} < 0.0001$ and $p_{adj} = 0.0336$, respectively) but not the hindbrain ($p_{adj} = 0.3316$) (Supplementary Figure S1). Raw Trp and Phe levels do not show significant differences except for raw Phe levels in the Telencephalon, which were higher in *rbfox1*$^{sa15940/sa15940}$ zebrafish ($p_{adj} = 0.0104$).

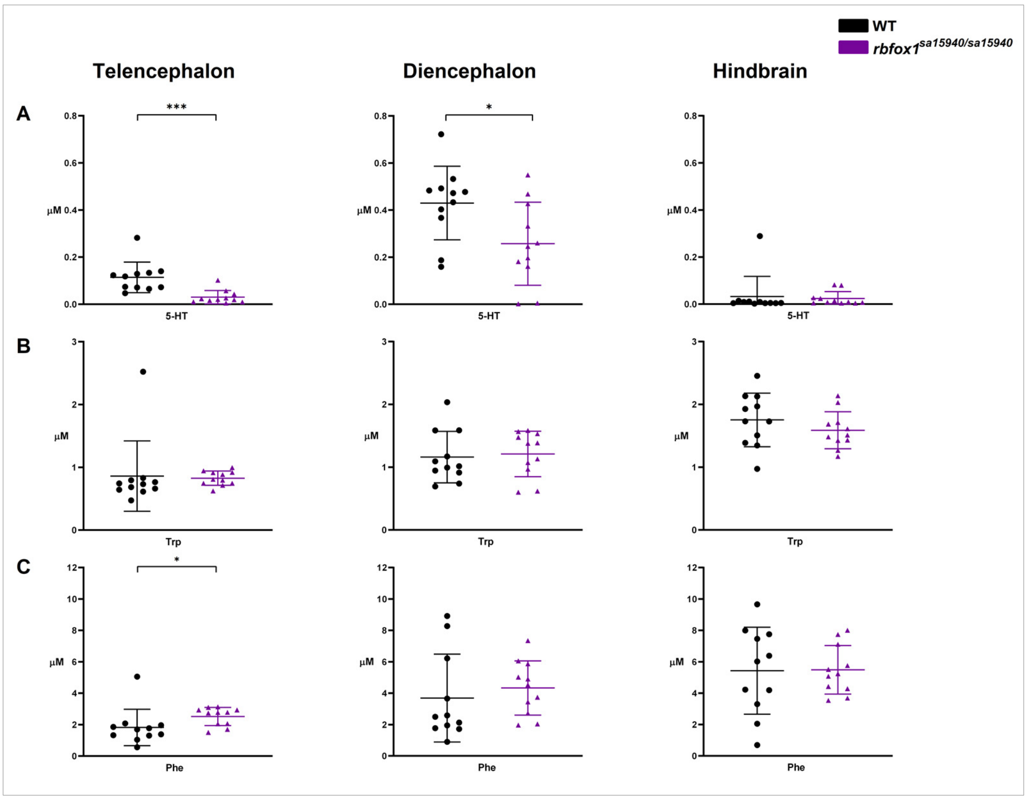

Supplementary Figure S1: Raw levels of serotonin, tryptophan, and phenylalanine in wildtype and *rbfox1*$^{sa15940/sa15940}$ zebrafish. (A) Serotonin (5-HT), **(B)** tryptophan (Trp) and **(C)** phenylalanine (Phe) levels of adult zebrafish were measured via ultra-high-performance liquid chromatography-tandem mass spectrometry (UPLC/MS-MS) in the telencephalon, diencephalon and hindbrain. WT = wildtype, *rbfox1*$^{sa15940/sa15940}$ = homozygous mutants, 5-HT = Serotonin, Trp = Tryptophan, Phe = Phenylalanine; N = 11 per group; Two tailed Mann-Whitney test; mean ± SD; *$p_{adj} < 0.05$, ***$p_{adj} < 0.001$.
